# Supplementary figures and images for: Liquid-Crystal Display (LCD) of achromatic, mean-modulated flicker in clinical assessment and experimental studies of visual systems
Source: PLoS One. 2021 Mar 24;16(3):e0248180. doi: 10.1371/journal.pone.0248180 (PMC7990305; doi:10.1371/journal.pone.0248180)

**A**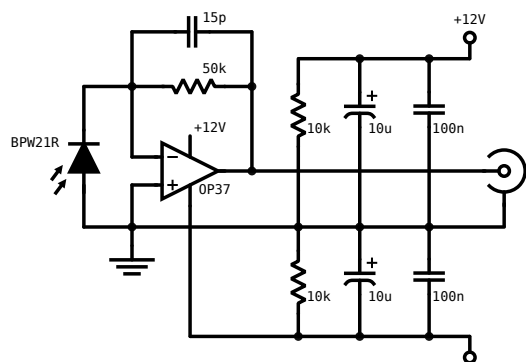**B**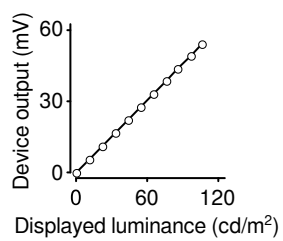**C**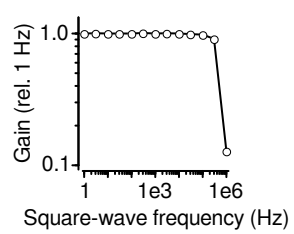

Supplement: S1 Fig — (PDF) [file pone.0248180.s001.pdf]
